# Anthracycline-Free Neoadjuvant Pertuzumab–Trastuzumab–Taxane in Patients with HER2-Positive Early Breast Cancer: Hormone Receptor Status as a Key Determinant of Pathological Complete Response

**DOI:** 10.3390/biomedicines14030717

**Published:** 2026-03-20

**Authors:** Azzurra Irelli, Tina Sidoni, Francesco Pavese, Silvia Rotondaro, Carla Luzi, Veronica Zelli, Sara Centonze, Leonardo Valerio Patruno, Francesca Zazzeroni, Alessandra Tessitore, Katia Cannita

**Affiliations:** 1 Medical Oncology Unit, Department of Oncology, Giuseppe Mazzini Hospital, 64100 Teramo, Italy; sara.centonze@aslteramo.it (S.C.); leonardovalerio.patruno@aslteramo.it (L.V.P.); katia.cannita@aslteramo.it (K.C.); 2 Medical Oncology, St. Salvatore Hospital, 67100 L’Aquila, Italy; tina_sidoni@yahoo.it; 3 Division of Gynecologic Oncology, Department of Woman and Child Health and Public Health, Fondazione Policlinico Universitario A. Gemelli IRCCS, 00168 Rome, Italy; francesco.pavese@outlook.it (F.P.); silviaroto@libero.it (S.R.); 4 Department of Biotechnological and Applied Clinical Sciences, University of L’Aquila, Via Vetoio, 67100 L’Aquila, Italy; carla.luzi@univaq.it (C.L.); veronica.zelli@univaq.it (V.Z.); francesca.zazzeroni@univaq.it (F.Z.); alessandra.tessitore@univaq.it (A.T.)

**Keywords:** HER2-positive breast cancer, neoadjuvant therapy, pathological complete response, pertuzumab, *PIK3CA*

## Abstract

**Background**: Neoadjuvant chemotherapy plus dual HER2 blockade is standard for HER2-positive early breast cancer (EBC), but the impact of hormone receptor (HR) status and *PIK3CA* mutations with anthracycline-free regimens remains unclear. **Methods**: We retrospectively analyzed 56 patients with stage II–III HER2-positive EBC treated with neoadjuvant pertuzumab–trastuzumab–taxane (THP) at a single institution. Pathological complete response (pCR, ypT0/is ypN0) was the primary endpoint; secondary endpoints were safety and early disease-free/overall survival (DFS/OS), while associations of HR status and *PIK3CA* mutations with pCR were explored. **Results**: The overall pCR rate was 60.7%, in line with major dual-HER2 neoadjuvant trials. HR-negative patients achieved higher pCR rates than HR-positive patients (85.7% vs. 45.7%; *p* = 0.007; odds ratio 7.125), identifying HR status as the main clinical factor associated with response. Among 36 patients with *PIK3CA* testing, pCR rates appeared similar in mutated and wild-type tumors (62.5% vs. 60.7%), but the small number of mutated cases precludes firm conclusions. At a median follow-up of 42 months, only five DFS and one OS event had occurred, so survival analyses are exploratory and should be interpreted cautiously. THP demonstrated an excellent safety profile, with minimal grade 3–4 toxicity, and no clinically relevant hematological, cardiac, or pulmonary events. **Conclusions**: Anthracycline-free THP is a highly active, well-tolerated neoadjuvant option for HER2-positive EBC, with particularly high pCR rates in HR-negative disease. HR status emerged as a key determinant of pCR, whereas the role of *PIK3CA* mutations remains inconclusive and requires confirmation in larger prospective studies.

## 1. Introduction

Neoadjuvant chemotherapy is a key component of treatment for early breast cancer (EBC), particularly in HER2-positive tumors, where it allows both local disease control and early eradication of micrometastases. Pathological complete response (pCR), defined as the absence of residual invasive disease in the breast and axillary lymph nodes (ypT0/is ypN0), is recognized as an important prognostic marker, especially in the HER2-positive and triple-negative settings. The CTNeoBC pooled analysis of more than 12,000 patients showed a strong association between pCR and long-term clinical benefit, while leaving open the debate on the value of pCR as a definitive surrogate endpoint for event-free survival (EFS) and overall survival (OS) [1].

The most recent international guidelines, including the 2024 ESMO recommendations, support neoadjuvant treatment with 6–8 cycles of chemotherapy combined with dual anti-HER2 blockade (trastuzumab plus pertuzumab) in patients with tumors clinically ≥cT2 and/or node-positive disease (≥cN1), with progressive extension of this approach to smaller, high-risk tumors (cT1c). Pivotal studies established this standard: the NOAH trial showed that adding trastuzumab to neoadjuvant chemotherapy doubled the pCR rate (from 19% to 38%) and significantly improved EFS, while subsequent escalation strategies testing various single- or dual-HER2 blockade combinations, demonstrated progressive improvements in pCR rates and survival outcomes [2].

Dual HER2 blockade with pertuzumab and trastuzumab combined with taxane-based chemotherapy has become the standard neoadjuvant approach for HER2-positive EBC, with pCR rates strongly influenced by hormone receptor (HR) status: median pCR rates of approximately 45% in HR-positive/HER2-positive tumors versus 70% in HR-negative/HER2-positive disease [3]. The NeoSphere trial [4,5] demonstrated that adding pertuzumab to trastuzumab plus docetaxel increases pCR rates from 29% to 46%, with a 5-year benefit in EFS. Subsequent trials (TRYPHAENA [6], TRAIN-2 [7], KRISTINE [8], NEOPETRA [9], GeparSepto [10]) and real-world studies (BrUOG [11], NEOPERSUR [12]) have consistently shown that anthracycline-free regimens combining dual HER2 blockade with taxanes achieve pCR rates of 60–70% in HR-negative disease and 45–50% in HR-positive disease, with significantly reduced hematological and cardiac toxicity compared with anthracycline-containing regimens. De-escalation strategies (WSG-ADAPT [13], neoCARHP [14], CompassHER2 pCR [15], PHERGain [16]) have further demonstrated that chemotherapy intensity can be reduced while maintaining high pCR rates and excellent survival outcomes, particularly in HR-negative patients.

In parallel, the role of *PIK3CA* mutations in HER2-positive tumors has emerged as an important but still controversial biological factor. *PIK3CA* mutations, present in approximately 20% of HER2-positive BCs—particularly in hotspot regions in exons 9 and 20—have been associated in several studies with lower pCR rates under anti-HER2 therapy, especially in HR-positive tumors, suggesting a potential role in partial resistance to HER2 blockade. However, the impact of these mutations on DFS and OS appears less clear, with inconsistent results across different series. Recent analyses in large prospective cohorts have confirmed the association between *PIK3CA* mutations and a lower likelihood of achieving pCR, but without a consistent worsening of long-term outcomes across all populations [17,18,19,20].

In this context of highly effective dual HER2 blockade with taxanes, progressive chemotherapy de-escalation and increasing attention to biomarker-driven personalization, it remains unclear whether, in HER2-positive tumors treated with modern neoadjuvant regimens (often anthracycline-free), *PIK3CA* mutations still exert an adverse effect on pCR and survival outcomes, and how this impact is modulated by HR status. The present study addresses this question by evaluating, in a real-world cohort treated with pertuzumab–trastuzumab–taxane (THP), the interplay between HR status, *PIK3CA* mutations, pCR rates, early survival outcomes and toxicity profile, with the aim of informing more effective and potentially de-escalated treatment strategies for HER2-positive EBC.

## 2. Materials and Methods

### 2.1. Study Population

This observational study, with a median follow-up of 42 months, aimed to evaluate the efficacy and safety of the THP regimen in patients with stage II–III HER2-positive BC.

Between April 2017 and March 2021, 56 consecutive patients with EBC completed pre-planned neoadjuvant systemic therapy followed by definitive breast surgery at the Medical Oncology Unit of San Salvatore Hospital, University of L’Aquila (Italy).

Eligibility criteria included: age ≥18 years; histologically confirmed HER2-positive BC, defined as IHC 3+ or IHC 2+ with FISH amplification according to local diagnostic procedures; early-stage disease treated with six cycles of pertuzumab (loading dose 840 mg, then 420 mg) and trastuzumab (loading dose 8 mg/kg, then 6 mg/kg) in combination with docetaxel (75 mg/m^2^) every 21 days.

Tumor response to neoadjuvant therapy was assessed using standardized imaging, including baseline ultrasound and reassessment every three cycles, plus mammography and/or magnetic resonance imaging at baseline and at the end of treatment.

Surgery was performed 3–6 weeks after the last cycle of neoadjuvant therapy.

The primary endpoint was the total pCR rate, defined as the absence of residual invasive disease in the breast and lymph nodes (ypT0/is ypN0), independently assessed by two experienced pathologists at the center.

Secondary endpoints included the incidence and severity of treatment-related adverse events (AEs), prospectively recorded according to NCI-CTC version 5, and graded as 1–2 or 3–4. Safety monitoring comprised routine laboratory tests at each cycle, including serial measurements of cardiac biomarkers (troponin I and NT-proBNP), as well as echocardiographic and electrocardiographic assessments every three cycles.

An exploratory ancillary analysis was performed to investigate the association between treatment efficacy and *PIK3CA* mutational status, evaluated on diagnostic biopsy samples available in a subgroup of 36 patients.

All living participants provided informed consent at the time of data collection. The study was conducted in accordance with Good Clinical Practice and the ethical principles of the Declaration of Helsinki and was approved by the local Ethics Committee of the L’Aquila center (protocol 32865/2018). Data collection was completed in December 2025.

### 2.2. PIK3CA Genotyping

*PIK3CA* genotyping was performed using an allele-specific real-time PCR assay. DNA was extracted from formalin-fixed paraffin-embedded (FFPE) tissue samples with the Zymo Quick DNA FFPE Kit (Zymo Research, Irvine, CA, USA), strictly following the manufacturer’s instructions.

Subsequently, mutational analysis was carried out using the EasyPGX *PIK3CA* kit (Diatech Pharmacogenetics, Jesi, Italy), a CE-IVD-certified assay, according to the manufacturer’s protocol. These highly sensitive assays, with limits of detection between 0.5% and 2%, enabled identification of approximately 90% of the known *PIK3CA* variants described in BC, based on data from the COSMIC database (Wellcome Sanger Institute, Hinxton, UK; accessed 12 December 2021). The analyses targeted specific hotspot regions in exons 4, 7, 9 and 20.

DNA quality and quantity were assessed using spectrophotometric measurements, and samples with DNA concentration < 10 ng/µL or A260/A280 ratio outside the range of 1.8–2.0 were excluded from further analysis. Only samples with adequate DNA integrity and purity were included in the *PIK3CA* mutational analysis to ensure reliability of the results.

### 2.3. Statistical Analysis

Baseline patient characteristics were summarized using descriptive statistics (means, medians, and proportions), and categorical variables were compared between groups using the chi-square test or Fisher’s exact test, as appropriate.

To identify independent predictors of pCR, we fitted a multivariable logistic regression model including HR status, tumor grade (2 vs. 3), and Ki-67 proliferation index (<20% vs. ≥20%) as covariates, selected based on clinical relevance and univariate associations with pCR. Additional clinical and pathological variables (age, menopausal status, and clinical T and N stage) were explored in an extended model but were not retained in the final parsimonious model because they were not statistically significant and their inclusion risked overfitting given the limited sample size (56 patients, 34 pCR events). *PIK3CA* mutational status was not included in the primary model because testing was available in only 36 patients (64% of the cohort); instead, an exploratory model including *PIK3CA* was fitted in this subset. Odds ratios (ORs) and 95% confidence intervals (CIs) were reported, and a two-sided *p*-value < 0.05 was considered statistically significant. All analyses were performed using Python (version 3.x; Python Software Foundation, Wilmington, DE, USA) with the statsmodels library (version 0.14). Given the exploratory nature of the study and the modest cohort size (*n* = 56 overall; *n* = 36 with *PIK3CA* testing, including only 8 mutated cases), no formal sample size calculation was performed. The study was not powered to detect statistically significant differences in pCR rates by *PIK3CA* status, and all analyses related to *PIK3CA* mutations should be regarded as hypothesis-generating. Post hoc power calculations indicated that, with eight mutated cases, the study had <20% power to detect a 20-percentage-point difference in pCR rates between *PIK3CA*-mutated and wild-type tumors at a two-sided α of 0.05; therefore, the absence of a statistically significant association between *PIK3CA* status and pCR in this cohort should not be interpreted as evidence of no effect, and larger prospective studies are needed to clarify this relationship.

## 3. Results

### 3.1. Patient Characteristics

Baseline patient characteristics are summarized in Table 1. The cohort included 21 patients (37.5%) with HR-negative and 35 (62.5%) with HR-positive HER2-positive EBC, with a median age of 54 years (range 28–79). Most patients had ECOG performance status 0 and low comorbidity burden, and the majority presented with T2–T4 and node-positive disease, reflecting a predominantly locally advanced population. Histologically, invasive ductal carcinoma and high Ki-67 (≥20%) were predominant. No statistically significant differences in baseline characteristics were observed between HR-negative and HR-positive groups (all *p* > 0.05).

### 3.2. Primary Endpoint: Pathological Complete Response

Overall, 34 of 56 patients achieved pCR, corresponding to a rate of 60.7% (ypT0/is ypN0), consistent with major clinical trials evaluating dual HER2 blockade combined with taxane-based neoadjuvant chemotherapy.

In this cohort, HR status emerged as the main clinical predictor of pCR (Table 2).

HR-negative patients achieved a pCR rate of 85.7% compared with 45.7% in HR-positive patients (*p* = 0.007, chi-square test; odds ratio 7.125, 95% CI 1.87–27.15), corresponding to an approximately seven-fold higher likelihood of pCR and a 40-percentage-point absolute difference.

Among the 36 patients with available *PIK3CA* testing, 28 (77.8%) were wild-type and eight (22.2%) harbored a *PIK3CA* mutation. No statistically significant association between *PIK3CA* status and pCR was observed (Table 3): pCR rates were 60.7% in wild-type tumors and 62.5% in mutated tumors (*p* = 1.000, Fisher’s exact test), but this exploratory analysis was limited by the small number of mutated cases and should be considered hypothesis-generating rather than definitive.

Stratified analysis according to HR and *PIK3CA* status (Table 4) confirmed HR status as the predominant factor associated with pCR in this cohort. HR-negative patients achieved high pCR rates irrespective of *PIK3CA* status, whereas within HR-positive disease, *PIK3CA*-mutated tumors showed only a modest trend toward lower pCR rates compared with wild-type, in the context of very small subgroup sizes.

Detailed genomic characterization of *PIK3CA* variants in the eight mutated patients is reported in Table 5. Hotspot mutations in exons 4, 9 and 20 were identified, along with a rare exon 7 variant, but the limited sample size did not allow robust correlation between specific variants and pCR.

### 3.3. Multivariable Analysis of pCR Predictors

Multivariable logistic regression analysis was performed to identify independent predictors of pCR among clinically relevant variables (Table 6).

In a model including HR status, tumor grade, and Ki-67 proliferation index (n = 56), HR status emerged as the only statistically significant independent predictor of pCR. HR-positive disease was associated with significantly lower odds of achieving pCR compared with HR-negative disease (OR 0.15, 95% CI 0.03–0.62; *p* = 0.009), confirming the univariate findings. Tumor grade (grade 3 vs. grade 2: OR 0.97, 95% CI 0.27–3.47; *p* = 0.958) and Ki-67 status (≥20% vs. <20%: OR 1.25, 95% CI 0.34–4.55; *p* = 0.734) were not independently associated with pCR in the multivariable model.

An exploratory multivariable analysis including *PIK3CA* mutational status was performed in the subset of 36 patients with available testing; however, this analysis was underpowered (only eight mutated cases) and yielded no statistically significant associations beyond a borderline trend for HR status (OR 0.14, 95% CI 0.02–1.05; *p* = 0.056). *PIK3CA* mutation status was not associated with pCR after adjustment for HR status and other covariates (OR 0.80, 95% CI 0.11–5.70; *p* = 0.824). These findings should be interpreted with caution due to the limited sample size and wide confidence intervals.

### 3.4. Secondary Endpoint: Early Survival Outcomes

At a median follow-up of 42 months (range 1.7–101.1), only five disease progression events and one death had occurred in the entire cohort. Given this low event rate, DFS and OS data are immature, median estimates are unreliable, and survival outcomes are summarized descriptively using landmark rates in Table 7, Table 8 and Table 9. No statistically robust differences by pCR, HR or *PIK3CA* status could be demonstrated, and all subgroup comparisons should be interpreted with caution as exploratory hypothesis-generating data.

### 3.5. Secondary Endpoint: Safety and Tolerability

All 56 patients were evaluable for safety. The THP regimen showed an excellent safety and tolerability profile, with AEs predominantly of grade 1–2 (Table 10).

Gastrointestinal (GI) toxicity was the most frequent class of event: diarrhea occurred in 62.5% of patients and nausea in 37.5%, with only 3.6% experiencing grade 3 diarrhea and no grade 3–4 nausea or vomiting.

Dermatologic and musculoskeletal events, including rash/dermatitis, onycholysis, myalgia and peripheral neuropathy, were generally mild (grade 1–2), and no grade 3–4 hematologic toxicity, febrile neutropenia, leucopenia, or thrombocytopenia was observed. Cardiac safety was excellent, with only one mild cardiac event and no grade 3–4 cardiac events, clinical heart failure, or treatment-related cardiac deaths. Importantly, no cases of clinically significant pulmonary toxicity, such as interstitial pneumonitis or drug-related pulmonary embolism, were reported despite the use of docetaxel. Overall, the favorable safety profile allowed completion of planned treatment in most patients.

## 4. Discussion

This observational analysis of 56 consecutive patients with HER2-positive EBC treated with neoadjuvant THP confirms the efficacy, safety, and clinical utility of dual HER2 blockade combined with taxane-based chemotherapy in this setting. The overall pCR rate of 60.7% observed in this cohort is consistent with major randomized trials and real-world evidence; in particular, the NeoSphere trial [4,5] showed that adding pertuzumab to trastuzumab plus docetaxel increased pCR rates from 29.0% to 45.8%.

Subsequent trials using dual HER2 blockade in combination with different taxane-based regimens (TRAIN-2 [7], TRYPHAENA [6], KRISTINE [8], NEOPETRA [9], GeparSepto [10]) have consistently reported pCR rates between 55% and 67%, and the 60.7% rate observed in our cohort lies well within this range. This concordance supports the standardization of the THP regimen as a neoadjuvant treatment option for HER2-positive EBC.

A key finding of this analysis is the identification of HR status as the main predictor of pCR, independent of other clinico-pathological variables. HR-negative patients achieved pCR in 85.7% of cases, compared with 45.7% in HR-positive patients (*p* = 0.007), corresponding to an approximately seven-fold higher likelihood of pCR (OR 7.125; 95% CI 1.87–27.15), and this 40-percentage-point absolute difference clearly exceeds what is typically reported for other molecular or clinical factors.

This observation is consistent with extensive evidence showing that HR-negative HER2-positive BCs are more sensitive to cytotoxic chemotherapy and anti-HER2 therapies. The underlying biological rationale relates to reduced “crosstalk” between HER2 signaling and the estrogen receptor (ER) pathway in HR-negative tumors: in the absence of an alternative HR-mediated proliferative drive, HER2-positive/HR-negative BCs are more dependent on HER2 signaling for growth and therefore more vulnerable to dual HER2 blockade [22].

Multivariable logistic regression analysis confirmed HR status as the only independent predictor of pCR in this cohort, with HR-positive disease associated with an approximately 85% reduction in the odds of achieving pCR (OR 0.15, 95% CI 0.03–0.62; *p* = 0.009) after adjustment for tumor grade and Ki-67 proliferation index. Neither grade nor Ki-67 retained independent prognostic significance in the multivariable model, suggesting that HR status is the dominant biological determinant of response to dual HER2 blockade in this setting. This finding aligns with extensive literature demonstrating that HR-negative/HER2-positive tumors exhibit markedly higher chemosensitivity and anti-HER2 therapy responsiveness compared with their HR-positive counterparts [23]. The biological mechanisms underlying the superior response to dual HER2 blockade in HR-negative versus HR-positive disease are multifactorial and involve complex crosstalk between HER2 and ER signaling pathways [24].

In HR-positive/HER2-positive tumors, ER activation provides an alternative proliferative drive that can partially compensate for HER2 inhibition, resulting in relative resistance to anti-HER2 therapy. Bidirectional interactions between ER and HER2 signaling—including ligand-independent ER activation via HER2-mediated phosphorylation and upregulation of HER family receptors in response to endocrine therapy—create escape mechanisms that reduce treatment efficacy [22].

Conversely, in HR-negative/HER2-positive tumors, cells are more dependent on HER2 signaling for growth and survival, rendering them particularly sensitive to dual HER2 blockade. The absence of ER-mediated resistance mechanisms, combined with higher proliferative activity (reflected by higher Ki-67 and grade 3 histology, as observed in our cohort), enhances chemosensitivity and contributes to the markedly higher pCR rates observed in this subgroup. Furthermore, HR-negative tumors may exhibit greater immune activation and enhanced antibody-dependent cellular cytotoxicity (ADCC) in response to trastuzumab and pertuzumab, thereby further augmenting treatment efficacy [25].

These findings underscore the importance of considering HR status not merely as a descriptive variable but as a key biological determinant that shapes therapeutic response and should inform treatment decision-making and future de-escalation strategies.

The clinical implications are substantial: HR status should be carefully considered when counseling patients regarding their likelihood of response and expected prognosis. The 85.7% pCR rate observed in HR-negative disease approaches the highest values reported in the literature and may support therapeutic de-escalation strategies tailored specifically to this favorable-prognosis subgroup [18,26].

In our cohort, *PIK3CA* mutations were detected in 22.2% of tested patients, in line with reported frequencies in HER2-positive BC, and pCR rates appeared similar in mutated and wild-type tumors. However, this exploratory analysis was constrained by the small number of mutated cases and the lack of pre-specified statistical power and therefore cannot exclude a clinically relevant impact of *PIK3CA* on treatment response. The observation that all HR-negative patients with *PIK3CA*-mutated tumors achieved pCR is intriguing and suggests that, under potent dual HER2 blockade, HR-negative disease may remain highly chemo-sensitive even in the presence of PI3K pathway alterations, but this hypothesis requires validation in larger, biologically annotated series.

This unexpected result, although limited by small sample size, raises the hypothesis that potent dual HER2 blockade might partially overcome PI3K pathway alterations in HR-negative disease through near-complete suppression of HER2 signaling. However, this observation is hypothesis-generating and requires prospective validation in larger, biologically annotated cohorts before drawing mechanistic conclusions. It is particularly noteworthy that HR-negative patients with *PIK3CA*-mutant tumors achieved a 100% pCR rate (3/3) compared with rates between 40% and 52.6% in HR-positive mutant cases, reinforcing the notion that HR status is a stronger determinant of response than *PIK3CA* mutational status. This pattern suggests that, in HR-negative disease treated with dual anti-HER2 blockade, PI3K pathway alterations may not provide meaningful escape routes, unlike in HR-positive tumors where HR signaling offers an alternative proliferative drive.

Overall, these data support the use of anthracycline-free THP as a highly active neoadjuvant option for HER2-positive EBC in daily practice, particularly in HR-negative patients, without suggesting any immediate need for *PIK3CA*-based treatment selection in this setting. Nevertheless, given the retrospective, single-center design, the limited sample size and the exploratory nature of the biomarker analyses, molecular profiling should currently be viewed as complementary to, rather than replacing, established clinical decision-making until prospective validation becomes available [20].

The THP regimen showed excellent tolerability, with most AEs being grade 1–2 and very few grade 3–4 events, a particularly relevant finding given the combined use of three active agents. GI toxicity was the most frequent class of AEs (diarrhea 62.5%, nausea 37.5%), as expected with taxanes and targeted therapies, but severe GI toxicity was rare, with only two cases of grade 3 diarrhea and no grade 3–4 nausea or vomiting; dermatologic and musculoskeletal events (myalgia 26.8%, peripheral neuropathy 17.9%) were mainly grade 1–2 and thus generally manageable, and the absence of grade 3–4 hematologic toxicity—including no febrile neutropenia, leucopenia or thrombocytopenia—suggests a substantial clinical advantage over anthracycline-containing regimens.

Cardiac safety was excellent, with only one mild cardiac event (grade 1–2), no grade 3–4 events, no clinical heart failure and no treatment-related cardiac deaths, in sharp contrast with anthracycline-based neoadjuvant regimens, which are associated with an estimated 5–10% risk of clinically relevant cardiac dysfunction; this cardiac profile supports omitting anthracyclines from neoadjuvant treatment for HER2-positive BC, in line with evidence from the TRAIN-2 [7] and TRYPHAENA [6] trials.

At a median follow-up of 42 months, the low number of events (5 DFS, 1 OS) reflects the favorable prognosis of this intensively treated cohort. Although survival data are immature and preclude definitive conclusions, the event rate was numerically lower in HR-negative patients and in those achieving pCR, consistent with the known prognostic value of these factors. Longer follow-up is needed to assess whether the high pCR rates observed translate into sustained DFS and OS benefits.

The trend toward longer survival in patients with *PIK3CA*-mutant tumors compared with wild-type, although not statistically significant due to the small sample size, supports continued monitoring and extended follow-up to better clarify any time-dependent impact of *PIK3CA* mutations on disease course. This single-center observational study, conducted in a relatively small cohort (*n* = 56), has inherent limitations: the design entails potential selection and data-collection bias, and although follow-up duration is adequate for safety assessment, it remains relatively short to precisely define long-term survival outcomes.

Survival endpoints were not part of the original analysis plan and, given the very low event rate, the DFS/OS results reported in Table 7, Table 8 and Table 9 are descriptive only and cannot be considered definitive.

This study has several limitations that warrant acknowledgment. The retrospective, single-center design and relatively small cohort size (*n* = 56) limit the generalizability of the findings and increase the risk of selection bias. The absence of a control arm with anthracycline-containing regimens precludes direct comparison of efficacy and safety outcomes between anthracycline-free and anthracycline-based approaches. *PIK3CA* testing was available in only 36 patients (64% of the cohort), and with only eight mutated cases, the analysis of *PIK3CA* associations with pCR was substantially underpowered, as acknowledged in the Methods. The multivariable logistic regression analysis, while informative, was constrained by the limited number of events (34 pCR) and could not accommodate a comprehensive set of prognostic covariates without risk of overfitting; therefore, some potentially relevant variables (e.g., tumor size, nodal burden, menopausal status) could not be fully evaluated. Early DFS and OS analyses were immature due to the low number of events (five progressions, one death) and relatively short median follow-up, precluding definitive prognostic conclusions. Finally, as a real-world observational study without pre-specified hypotheses or formal statistical power calculations, all findings—particularly those related to *PIK3CA*—should be considered exploratory and hypothesis-generating rather than definitive.

Future prospective, multi-center studies with larger cohorts and pre-specified statistical power are needed to confirm the independent prognostic role of HR status and to definitively assess the impact of *PIK3CA* mutations on response to anthracycline-free dual HER2 blockade regimens. Integration of comprehensive genomic profiling, including additional resistance mechanisms beyond *PIK3CA* (e.g., *PTEN* loss, *ESR1* mutations), alongside functional imaging biomarkers (e.g., 18F-FDG PET) could further refine treatment selection and identify patients most likely to benefit from de-escalated or intensified strategies. Validation of these biomarkers in prospective trials with adaptive designs would represent a meaningful step toward precision medicine in HER2-positive EBC.

## 5. Conclusions

This observational analysis of neoadjuvant THP in 56 consecutive patients with HER2-positive EBC confirms the efficacy, safety, and clinical value of this therapeutic approach. These findings support the use of dual HER2 blockade with pertuzumab and trastuzumab combined with taxane-based neoadjuvant chemotherapy as a standard treatment.

In this real-world cohort, HR status, rather than *PIK3CA* mutational status, emerged as the main determinant of pCR under anthracycline-free THP, although the exploratory design and small biomarker-tested subset preclude definitive conclusions. While our findings do not indicate a clear negative effect of *PIK3CA* mutations on response, they should primarily be regarded as hypothesis-generating and as a rationale for future biomarker-driven de-escalation trials in HER2-positive EBC.

The excellent safety profile—especially the lack of clinically relevant hematological and cardiac toxicity—supports the omission of anthracyclines from neoadjuvant regimens in HER2-positive disease when dual anti-HER2 blockade is used, in line with recent de-escalation trial data.

Longer follow-up of this cohort will be essential to determine whether the high pCR rates, particularly in HR-negative patients, translate into sustained improvements in DFS and OS. It will also be important to explore additional molecular biomarkers of response and resistance beyond *PIK3CA* mutations to identify patients who may benefit most from treatment intensification or alternative therapeutic strategies; prospective studies incorporating real-time molecular profiling and adaptive trial designs could further optimize treatment selection and represent a step toward precision medicine in HER2-positive BC.

## Figures and Tables

**Table 1:** Comprehensive baseline demographic and clinical characteristics.

Characteristic	HR-Negative (*n* = 21)	HR-Positive (*n* = 35)	Overall (*n* = 56)
Age (years)			
Median (range)	52 (28–75)	54 (33–79)	54 (28–79)
<40 years, *n* (%)	3 (14.3%)	4 (11.4%)	7 (12.5%)
≥40 years, *n* (%)	18 (85.7%)	31 (88.6%)	49 (87.5%)
Menopausal status			
Premenopausal, n (%)	8 (38.1%)	14 (40.0%)	22 (39.3%)
Postmenopausal, *n* (%)	13 (61.9%)	21 (60.0%)	34 (60.7%)
ECOG Performance Status			
0, *n* (%)	17 (81.0%)	32 (91.4%)	49 (87.5%)
1, *n* (%)	3 (14.3%)	3 (8.6%)	6 (10.7%)
2, *n* (%)	1 (4.8%)	0 (0.0%)	1 (1.8%)
CIRS Comorbidity Index			
Stable (0), *n* (%)	13 (61.9%)	24 (68.6%)	37 (66.1%)
Intermediate (1), *n* (%)	5 (23.8%)	7 (20.0%)	12 (21.4%)
Secondary (2), *n* (%)	3 (14.3%)	4 (11.4%)	7 (12.5%)
Cardiovascular comorbidities			
Yes, *n* (%)	6 (28.6%)	9 (25.7%)	15 (26.8%)
No, *n* (%)	15 (71.4%)	26 (74.3%)	41 (73.2%)
ER/PgR status			
Negative, *n* (%)	21 (100.0%)	0 (0.0%)	21 (37.5%)
Positive, *n* (%)	0 (0.0%)	35 (100.0%)	35 (62.5%)
Histological classification			
Ductal, *n* (%)	19 (90.5%)	28 (80.0%)	47 (83.9%)
Lobular, *n* (%)	1 (4.8%)	6 (17.1%)	7 (12.5%)
Other, *n* (%)	1 (4.8%)	1 (2.9%)	2 (3.6%)
Tumor grade			
Grade 2, *n* (%)	10 (47.6%)	25 (71.4%)	35 (62.5%)
Grade 3, *n* (%)	11 (52.4%)	10 (28.6%)	21 (37.5%)
Ki-67 proliferation index			
<20%, *n* (%)	3 (14.3%)	13 (37.1%)	16 (28.6%)
≥20%, *n* (%)	18 (85.7%)	22 (62.9%)	40 (71.4%)
Clinical T stage			
T1, *n* (%)	3 (14.3%)	4 (11.4%)	7 (12.5%)
T2, *n* (%)	13 (61.9%)	24 (68.6%)	37 (66.1%)
T3, *n* (%)	4 (19.0%)	3 (8.6%)	7 (12.5%)
T4, *n* (%)	1 (4.8%)	4 (11.4%)	5 (8.9%)
Clinical N stage			
N0, *n* (%)	6 (28.6%)	7 (20.0%)	13 (23.2%)
N1, *n* (%)	12 (57.1%)	20 (57.2%)	32 (57.1%)
N2, *n* (%)	3 (14.3%)	8 (22.8%)	11 (19.7%)
N3, *n* (%)	0	0	0
Type of breast surgery			
Quadrantectomy, *n* (%)	4 (19.0%)	18 (51.4%)	22 (39.3%)
Mastectomy, *n* (%)	17 (81.0%)	17 (48.6%)	34 (60.7%)

Note: HR = hormone receptor. ECOG = Eastern Cooperative Oncology Group performance status. CIRS = Cumulative Illness Rating Scale. ER/PgR = estrogen/progesterone receptor. All patients had HER2-positive early-stage BC. No statistically significant differences between HR-negative and HR-positive groups (all *p* > 0.05).

**Table 2:** Pathologic complete response by HR status.

HR Status	pCR *n* (%)	Non-pCR *n* (%)	Total *n* (%)	pCR Rate (%)	OR (95% CI)	*p*-Value
HR-negative	18 (85.7%)	3 (14.3%)	21 (100%)	85.7%	7.125 (1.87–27.15)	0.007 **
HR-positive	16 (45.7%)	19 (54.3%)	35 (100%)	45.7%	Ref	
Overall	34 (60.7%)	22 (39.3%)	56 (100%)	60.7%		

Note: Chi-square test. ** Statistically significant at *p* < 0.05. OR = Odds Ratio; Ref = reference category (HR-positive). HR-negative patients demonstrated significantly superior pCR rates compared to HR-positive patients (absolute difference = 40 percentage points).

**Table 3:** Pathologic complete response by *PIK3CA* mutation status.

*PIK3CA* Status	pCR *n* (%)	Non-pCR *n* (%)	Total *n* (%)	pCR Rate (%)	OR (95% CI)	*p*-Value
*PIK3CA* Wild-type	17 (60.7%)	11 (39.3%)	28 (100%)	60.7%	0.927 (0.174–4.932)	1.000
*PIK3CA* Mutated	5 (62.5%)	3 (37.5%)	8 (100%)	62.5%	Ref	NS

Note: Fisher exact test. NS = not statistically significant (*p* > 0.05). OR = Odds Ratio. Analysis performed among 36 patients with *PIK3CA* testing available. 20 patients without *PIK3CA* testing excluded. pCR = pathologic complete response (ypT0/is ypN0). Despite reports in the literature of *PIK3CA*-mediated resistance in HER2-positive disease, no negative impact on pCR was observed in this THP-treated cohort.

**Table 4:** Pathologic complete response by HR status and *PIK3CA* mutation.

Subgroup	pCR *n*	Non-pCR *n*	Total *n*	pCR Rate (%)
HR-negative/*PIK3CA* WT	7	2	9	77.8%
HR-negative/*PIK3CA* mut	3	0	3	100.0%
HR-positive/*PIK3CA* WT	10	9	19	52.6%
HR-positive/*PIK3CA* mut	2	3	5	40.0%

Note: 36 patients with both HR and *PIK3CA* testing available. HR status remains the dominant predictor of pCR independent of *PIK3CA* mutation status. Within HR-positive disease, *PIK3CA* mutation trends toward lower pCR (52.6% vs. 40.0%), but sample size is limited (*n* = 5 mutated).

**Table 5:** Clinical features of *PIK3CA* mutant patients and variants detected.

Pt. ID	ER/PgR	Ki67	Pathological Response	N345x	C420R	E542x	E545x	Q546x	H1047x	G1049x
1	pos.	<20%	non-pCR	WT	WT	WT	MUT	WT	MUT	WT
2	pos.	<20%	pCR	WT	WT	WT	WT	MUT	WT	WT
3	neg.	≥20%	pCR	WT	WT	WT	WT	WT	MUT	WT
4	neg.	<20%	pCR	WT	WT	WT	WT	WT	MUT	WT
5	neg.	≥20%	pCR	WT	WT	WT	WT	WT	MUT	WT
6	pos.	≥20%	non-pCR	MUT	WT	WT	WT	WT	WT	WT
7	neg.	≥20%	pCR	WT	WT	WT	WT	WT	MUT	WT
8	pos.	≥20%	non-pCR	WT	WT	WT	WT	WT	MUT	WT

Variants as follows: c.1033A>C, c.1034A>T, c.1034A>C, c.1035C>A, indistinguishable (N345x, exon 4); c.1258T>C (C420R, exon 7); c.1624G>A, c.1625A>G, c.1625A>T, indistinguishable (E542x, exon 9); c.1633G>A, c.1633G>C, c.1634A>C, c.1634A>G, c.1635G>C, c.1635G>T, indistinguishable (E545x, exon 9); c.1636C>A, c.1636C>G, c.1637A>C, c.1637A>G, c.1637A>T, indistinguishable (Q546x, exon 9); c.3139C>T, c.3140A>G, c.3140A>T, indistinguishable (H1047x, exon 20); c.3145G>C, c.3146G>C, indistinguishable (G1049x, exon 20). Note: Detailed genomic analysis of *PIK3CA* mutations detected in eight patients with available testing. N345x, E542x, E545x, and Q546x represent hotspot mutations in exons 4 and 9 (helical domain). H1047x represents hotspot mutations in exon 20 (kinase domain). C420R is a rare variant in exon 7. MUT = mutated; WT = wild-type. Patients 1, 6, and 8 (all HR-positive, non-pCR) harbored exon 9 or 20 hotspot mutations. Notably, all HR-negative patients with *PIK3CA* mutations (3, 4, 5, 7) achieved pCR, suggesting superior response to dual HER2 blockade in HR-negative disease regardless of PI3K pathway alterations. Pt. ID = patient identifier; ER/PgR = estrogen/progesterone receptor; pos. = positive; neg. = negative; pCR = pathologic complete response. Part of the content of this table has been adapted and updated from: Irelli A., et al. (2022), published under the Creative Commons Attribution (CC BY 4.0) license [21].

**Table 6:** Multivariable logistic regression analysis of pathological complete response predictors (*n* = 56).

Variable	OR	95% CI	*p*-Value
HR-positive (vs. HR-negative)	0.15	0.03–0.62	0.009 **
Grade 3 (vs. Grade 2)	0.97	0.27–3.47	0.958
Ki-67 ≥ 20% (vs. <20%)	1.25	0.34–4.55	0.734

Note: Multivariable logistic regression model including HR status, tumor grade, and Ki-67 proliferation index as independent variables. OR = Odds Ratio; CI = Confidence Interval. ** Statistically significant at *p* < 0.05. HR-positive status independently predicts lower probability of achieving pCR, consistent with univariate analysis (Table 2). Grade and Ki-67 did not reach statistical significance after adjustment for HR status. Model statistics: AIC = 73.37, Pseudo R^2^ = 0.129, LLR *p*-value = 0.022.

**Table 7:** Disease-free survival and overall survival by pCR status.

Outcome	pCR (*n* = 34)	Non-pCR (*n* = 22)	Median Difference	*p*-Value	Statistical Test
DFS (months)					
Median (range)	42.0 (1.7–95.6)	37.7 (6.0–101.1)	+4.3 months	0.808	Mann–Whitney U
Events (PD), *n/N*	2/34	3/22			
Event rate (%)	5.9%	13.6%			
OS (months)					
Median (range)	42.0 (2.7–95.6)	42.1 (12.2–101.1)	−0.1 months	0.540	Mann–Whitney U
Events (death), *n/N*	1/34	0/22			
Event rate (%)	2.9%	0.0%			

Note: Follow-up median ~42 months (range 1.7–101.1 months). Early event rate (5 DFS events, 1 OS event) limits definitive prognostic assessment. Longer follow-up needed to evaluate pCR as prognostic marker in THP cohort. Exploratory descriptive analysis; survival endpoints were not pre-specified or statistically powered. With only five DFS events and one OS event, median estimates are unreliable and provided for descriptive purposes only. Definitive survival comparisons require longer follow-up.

**Table 8:** Disease-free survival and overall survival by HR status.

Outcome	HR-Negative (*n* = 21)	HR-Positive (*n* = 35)	Median Difference	*p*-Value	Statistical Test
DFS (months)					
Median (range)	49.8 (6.0–95.6)	31.5 (1.7–101.1)	+18.3 months	0.456	Mann–Whitney U
Events (PD), *n/N*	1/21	4/35			
Event rate (%)	4.8%	11.4%			
OS (months)					
Median (range)	49.8 (16.1–95.6)	35.0 (2.7–101.1)	+14.8 months	0.520	Mann–Whitney U
Events (death), *n/N*	0/21	1/35			
Event rate (%)	0.0%	2.9%			

Note: HR-negative patients show numerically longer median DFS (+18.3 months) and OS (+14.8 months), though differences do not reach statistical significance, likely due to low event rate. Exploratory descriptive analysis; survival endpoints were not pre-specified or statistically powered. With only five DFS events and one OS event, median estimates are unreliable and provided for descriptive purposes only. Definitive survival comparisons require longer follow-up.

**Table 9:** Disease-free survival and overall survival by *PIK3CA* status.

Outcome	*PIK3CA* WT (*n* = 28)	*PIK3CA* Mut (*n* = 8)	Median Difference	*p*-Value	Statistical Test
DFS (months)					
Median (range)	32.5 (1.7–101.1)	66.2 (12.2–72.9)	−33.7 months	0.195	Mann–Whitney U
Events (PD), *n/N*	3/28	1/8			
Event rate (%)	10.7%	12.5%			
OS (months)					
Median (range)	39.5 (2.7–101.1)	66.2 (12.2–72.9)	−26.7 months	0.322	Mann–Whitney U
Events (death), *n/N*	1/28	0/8			
Event rate (%)	3.6%	0.0%			

Note: Analysis among 36 patients with *PIK3CA* testing available. Unexpectedly, *PIK3CA*-mutated tumors show trend toward longer median DFS (−33.7 months favoring mutation) and OS (−26.7 months), but differences are not statistically significant and sample size limited (n = 8 mutated). This contradicts prior prognostic models and may reflect effectiveness of dual HER2 blockade in overcoming PI3K resistance. Exploratory descriptive analysis; survival endpoints were not pre-specified or statistically powered. With only five DFS events and one OS event, median estimates are unreliable and provided for descriptive purposes only. Definitive survival comparisons require longer follow-up.

**Table 10:** Treatment-related adverse events (CTCAE v.5.0) during neoadjuvant therapy.

Adverse Event	Any Grade *n* (%)	Grade 1–2 *n* (%)	Grade 3–4 *n* (%)
Nausea	21 (37.5%)	21 (37.5%)	0 (0.0%)
Vomiting	2 (3.6%)	2 (3.6%)	0 (0.0%)
Diarrhea	35 (62.5%)	33 (58.9%)	2 (3.6%)
Stomatitis	15 (26.8%)	15 (26.8%)	0 (0.0%)
Rash/Dermatitis	2 (3.6%)	2 (3.6%)	0 (0.0%)
Onycholysis	10 (17.9%)	10 (17.9%)	0 (0.0%)
Myalgia	15 (26.8%)	15 (26.8%)	0 (0.0%)
Peripheral neuropathy	10 (17.9%)	10 (17.9%)	0 (0.0%)
Anemia	5 (8.9%)	5 (8.9%)	0 (0.0%)
Leukopenia/Neutropenia	0 (0.0%)	0 (0.0%)	0 (0.0%)
Thrombocytopenia	0 (0.0%)	0 (0.0%)	0 (0.0%)
Cardiac toxicity	1 (1.8%)	1 (1.8%)	0 (0.0%)
Pulmonary toxicity	0 (0.0%)	0 (0.0%)	0 (0.0%)

Note: All 56 patients were evaluable for safety. CTCAE v5.0 grading used throughout study. Most common AEs were gastrointestinal (diarrhea 62.5%, nausea 37.5%) and myalgia (26.8%). Grade 3–4 toxicity was minimal with only two Grade 3 diarrhea cases; no Grade 3–4 hematologic toxicity, no clinical cardiac events, and no treatment-related deaths. Overall, THP demonstrated excellent tolerability with predominantly mild-to-moderate toxicity.

## Data Availability

The data supporting the findings of this study are not publicly available because participants did not provide consent for their individual data to be shared and due to applicable institutional and national data protection regulations.

## Abbreviations

The following abbreviations are used in this manuscript:

ADCC	Antibody-Dependent Cellular Cytotoxicity
AE	Adverse Event
BC	Breast cancer
BrUOG	Brown University Oncology Research Group
CIRS	Cumulative Illness Rating Scale (CIRS)
CompassHER2	COMprehensive use of Pathologic response ASSessment to optimize therapy in HER2-positive breast cancer
CTNeoBC	Clinical Trials Network for Neoadjuvant Breast Cancer
DFS	Disease-Free Survival
EBC	Early Breast Cancer
ECOG	Eastern Cooperative Oncology Group
EFS	Event-Free Survival
ER	Estrogen Receptor
ESMO	European Society for Medical Oncology
FISH	Fluorescence In Situ Hybridization
GI	Gastrointestinal
HR	Hormone receptor
HER2	Human Epidermal Growth Factor Receptor 2
IC	Confidence Interval
IHC	Immunohistochemistry
NCI-CTC	National Cancer Institute Common Terminology Criteria
NOAH	NeOAdjuvant Herceptin
NT-proBNP	N-terminal pro-B-type Natriuretic Peptide
ORR	Objective Response Rate
OS	Overall Survival
pCR	Pathological Complete Response
THP	Pertuzumab–trastuzumab–taxane
PET	Positron Emission Tomography
PI3K	Phosphatidylinositol 3-Kinase
PIK3CA	Phosphatidylinositol-4,5-bisphosphate 3-kinase catalytic subunit alpha
PR	Progesterone Receptor
PS	Performance Status
RFS	Recurrence-Free Survival
THP	Pertuzumab-Trastuzumab-Taxane
WSG-ADAPT	West German Study Group- Adjuvant Dynamic marker-Adjusted Personalized Therapy

## References

[1] Cortazar P., Zhang L., Untch M., Mehta K., Costantino J.P., Wolmark N., Bonnefoi H., Cameron D., Gianni L., Valagussa P. (2014). Pathological complete response and long-term clinical benefit in breast cancer: The CTNeoBC pooled analysis. Lancet.

[2] Loibl S., André F., Bachelot T., Barrios C.H., Bergh J., Burstein H.J., Cardoso M.J., Carey L.A., Dawood S., Del Mastro L. (2024). Early breast cancer: ESMO Clinical Practice Guideline for diagnosis, treatment and follow-up. Ann. Oncol..

[3] Harbeck N., Gluz O. (2017). Neoadjuvant therapy for triple negative and HER2-positive early breast cancer. Breast.

[4] Gianni L., Pienkowski T., Im Y.H., Roman L., Tseng L.M., Liu M.C., Lluch A., Staroslawska E., de la Haba-Rodriguez J., Im S.A. (2012). Efficacy and safety of neoadjuvant pertuzumab and trastuzumab in women with locally advanced, inflammatory, or early HER2-positive breast cancer (NeoSphere): A randomised multicentre, open-label, phase 2 trial. Lancet Oncol..

[5] Gianni L., Pienkowski T., Im Y.H., Tseng L.M., Liu M.C., Lluch A., Starosławska E., de la Haba-Rodriguez J., Im S.A., Pedrini J.L. (2016). 5-year analysis of neoadjuvant pertuzumab and trastuzumab in patients with locally advanced, inflammatory, or early-stage HER2-positive breast cancer (NeoSphere): A multicentre, open-label, phase 2 randomised trial. Lancet Oncol..

[6] Schneeweiss A., Chia S., Hickish T., Harvey V., Eniu A., Hegg R., Tausch C., Seo J.H., Tsai Y.F., Ratnayake J. (2013). Pertuzumab plus trastuzumab in combination with standard neoadjuvant anthracycline-containing and anthracycline-free chemotherapy regimens in patients with HER2-positive early breast cancer: A randomized phase II cardiac safety study (TRYPHAENA). Ann Oncol..

[7] van Ramshorst M.S., van der Voort A., van Werkhoven E.D., Mandjes I.A., Kemper I., Dezentjé V.O., Oving I.M., Honkoop A.H., Tick L.W., van de Wouw A.J. (2018). Neoadjuvant chemotherapy with or without anthracyclines in the presence of dual HER2 blockade for HER2-positive breast cancer (TRAIN-2): A multicentre, open-label, randomised, phase 3 trial. Lancet Oncol..

[8] Hurvitz S.A., Martin M., Jung K.H., Huang C.S., Harbeck N., Valero V., Stroyakovskiy D., Wildiers H., Campone M., Boileau J.F. (2019). Neoadjuvant Trastuzumab Emtansine and Pertuzumab in Human Epidermal Growth Factor Receptor 2-Positive Breast Cancer: Three-Year Outcomes From the Phase III KRISTINE Study. J. Clin. Oncol..

[9] González-Santiago S., Saura C., Ciruelos E., Alonso J.L., de la Morena P., Eslava M.S., Sancho M.I.G., de Luna A., Dalmau E., Servitja S. (2020). Real-world effectiveness of dual HER2 blockade with pertuzumab and trastuzumab for neoadjuvant treatment of HER2-positive early breast cancer (The NEOPETRA Study). Breast Cancer Res. Treat..

[10] Loibl S., Jackisch C., Schneeweiss A., Schmatloch S., Aktas B., Denkert C., Wiebringhaus H., Kümmel S., Warm M., Paepke S. (2017). Dual HER2-blockade with pertuzumab and trastuzumab in HER2-positive early breast cancer: A subanalysis of data from the randomized phase III GeparSepto trial. Ann. Oncol..

[11] Lopresti M.L., Bian J.J., Sakr B.J., Strenger R.S., Legare R.D., Fenton M., Witherby S.M., Dizon D.S., Pandya S.V., Stuckey A.R. (2021). Neoadjuvant weekly paclitaxel and carboplatin with trastuzumab and pertuzumab in HER2-positive breast cancer: A Brown University Oncology Research Group (BrUOG) study. Breast Cancer Res. Treat..

[12] Falcón González A., Jurado J.C., Valenti E.L., Cubero R.U., de la Gala M.C.A., Guisado M.A.M., Ambite R.A., González C.J.R., Góngora M.A., Pérez L.R. (2024). Real-world experience with pertuzumab and trastuzumab combined with chemotherapy in neoadjuvant treatment for patients with early-stage HER2-positive breast cancer: The NEOPERSUR study. Clin. Transl. Oncol..

[13] Nitz U.A., Gluz O., Christgen M., Grischke E.M., Augustin D., Kuemmel S., Braun M., Potenberg J., Kohls A., Krauss K. (2017). De-escalation strategies in HER2-positive early breast cancer (EBC): Final analysis of the WSG-ADAPT HER2+/HR- phase II trial: Efficacy, safety, and predictive markers for 12 weeks of neoadjuvant dual blockade with trastuzumab and pertuzumab ± weekly paclitaxel. Ann. Oncol..

[14] Gao H.F., Wei L., Wu Z., Dong J., Cao Y., Zhao Y., Chen Q.J., Ma S., Ouyang J., Ye J.H. (2025). De-escalated neoadjuvant taxane plus trastuzumab and pertuzumab with or without carboplatin in HER2-positive early breast cancer (neoCARHP): A multicentre, open-label, randomised, phase 3 trial. J. Clin. Oncol..

[15] Tung N., Zhao F., DeMichele A., Prat A., Winer E., Wright J., Recht A., Weiss A., Tjoe J., Feldman S. (2025). Predicting pathologic complete response (pCR) from clinicopathologic variables and HER2DX genomic test in stage II/III HER2+ breast cancer treated with taxane, trastuzumab, and pertuzumab (THP): Secondary results from the EA1181/CompassHER2 pCR trial. J. Clin. Oncol..

[16] Pérez-García J.M., Cortés J., Ruiz-Borrego M., Colleoni M., Stradella A., Bermejo B., Dalenc F., Escrivá-de-Romaní S., Martínez L.C., Ribelles N. (2024). 3-Year Invasive Disease-Free Survival with Chemotherapy de-Escalation Using an 18F-FDG-PETBased, Pathological Complete Response-Adapted Strategy in HER2-Positive Early Breast Cancer (PHERGain): A Randomised, Open-Label, Phase 2 Trial. Lancet.

[17] Loibl S., von Minckwitz G., Schneeweiss A., Paepke S., Lehmann A., Rezai M., Zahm D.M., Sinn P., Khandan F., Eidtmann H. (2014). PIK3CA mutations are associated with lower rates of pathologic complete response to anti-human epidermal growth factor receptor 2 (her2) therapy in primary HER2-overexpressing breast cancer. J. Clin. Oncol..

[18] Goel S., Krop I.E. (2016). PIK3CA mutations in HER2-positive breast cancer: An ongoing conundrum. Ann. Oncol..

[19] Fan H., Li C., Xiang Q., Xu L., Zhang Z., Liu Q., Zhang T., Zhou Y., Zhao X., Cui Y. (2018). PIK3CA mutations and their response to neoadjuvant treatment in early breast cancer: A systematic review and meta-analysis. Thorac. Cancer.

[20] Jank P., Karn T., van Mackelenbergh M., Lindner J., Treue D., Huober J., Engels K., Solbach C., Diebold K., Marmé F. (2024). An Analysis of PIK3CA Hotspot Mutations and Response to Neoadjuvant Therapy in Patients with Breast Cancer from Four Prospective Clinical Trials. Clin. Cancer Res..

[21] Irelli A., Parisi A., D’Orazio C., Sidoni T., Rotondaro S., Patruno L., Pavese F., Bafile A., Resta V., Pizzorno L. (2022). Anthracycline-Free Neoadjuvant Treatment in Patients with HER2-Positive Breast Cancer: Real-Life Use of Pertuzumab, Trastuzumab and Taxanes Association with an Exploratory Analysis of PIK3CA Mutational Status. Cancers.

[22] Pegram M., Jackisch C., Johnston S.R.D. (2023). Estrogen/HER2 receptor crosstalk in breast cancer: Combination therapies to improve outcomes for patients with hormone receptor-positive/HER2-positive breast cancer. NPJ Breast Cancer.

[23] Liu S., Yu M., Mou E., Wang M., Liu S., Xia L., Li H., Tang H., Feng Y., Yu X. (2025). The optimal neoadjuvant treatment strategy for HR+/HER2 + breast cancer: A network meta-analysis. Sci. Rep..

[24] Arpino G., Wiechmann L., Osborne C.K., Schiff R. (2008). Crosstalk between the estrogen receptor and the HER tyrosine kinase receptor family: Molecular mechanism and clinical implications for endocrine therapy resistance. Endocr. Rev..

[25] Tsao L.C., Crosby E.J., Trotter T.N., Wei J., Wang T., Yang X., Summers A.N., Lei G., Rabiola C.A., Chodosh L.A. (2022). Trastuzumab/pertuzumab combination therapy stimulates antitumor responses through complement-dependent cytotoxicity and phagocytosis. JCI Insight.

[26] Fan H., Li C., Xiang Q., Xu L., Zhang Z., Liu Q., Zhang T., Zhou Y., Zhao X., Cui Y. (2021). PIK3CA mutations and the response to anti-HER2 therapy in breast cancer. Cancer Cell Int..

